# Correction: A systematic review and meta-analysis of antimicrobial resistance knowledge, attitudes, and practices: Current evidence to build a strong national antimicrobial drug resistance narrative in Ethiopia

**DOI:** 10.1371/journal.pone.0337394

**Published:** 2025-11-20

**Authors:** Beshada Zerfu Woldegeorgis, Amene Abebe Kerbo, Mohammed Suleiman Obsa, Taklu Marama Mokonnon

There is an error in Table 1 in row 5. The region name was incorrectly reported as “Amahra.” The correct region name should be “Oromia.”

**Table 1:** The characteristics of the studies included in the systematic review and meta-analysis.

S.No	Author	Year	Region	Study design	Sample size	Good knowledge, n (%)	Favorable attitudes, n (%)	Good practices, n (%)	Response rate	Appraisal score
1	Bayeh et *al*.[39]	2014	Amhara	Cross-sectional	385	278 (72.21)	NR	NR	91.4%	9
2	Belachew et *al*.[38]	2022	Amhara	Cross-sectional	276	210 (76.09)	198 (71.74)	181 (65.58)	86.6%	9
3	Beyene et *al.*[37]	2017	Addis Ababa	Cross-sectional	205	NR	195 (95.12)	NR	NR	9
4	Dejene et *al.*[36]	2022	Amhara	Cross-sectional	400	180 (45.00)	215 (53.75)	200 (50.00)	NR	9
5	Fetensa et *al.*[35]	2020	Oromia	Cross-sectional	232	158 (64.10)	NR	NR	93.6%	9
6	Gebeyehu et *al.[27]*	2021	Amhara	Cross-sectional	571	110 (19.26)	84 (14.71)	NR	100%	8
7	Gemeda et *al.[34]*	2020	Amhara and Oromia	Cross-sectional	379	70 (18.47)	263 (69.39)	NR	NR	9
8	Geta and Kibret[33]	2021	Amhara	Cross-sectional	91	46 (50.55)	48 (52.75)	43 (47.25)	100%	9
9	Geta and Kibret[32]	2022	Amhara	Cross-sectional	232	87 (37.50)	105 (45.26)	102 (43.97)	NR	9
10	Mengesha et *al.*[31]	2020	Amhara	Cross-sectional	374	160 (42.78)	194 (50.52)	NR	97.0%	8
11	Seid et *al.*[30]	2018	Amhara	Cross-sectional	323	145 (44.89)	311 (96.28)	NR	90.0%	9
12	Simegn et *al.*[26]	2022	Amhara	Cross-sectional	412	349 (84.71)	NR	NR	97.4%	8
13	Tafa et *al.*[29]	2017	Dire Dawa	Cross-sectional	218	137 (62.84)	184 (84.40)	NR	NR	8
14	Tesfaye et *al.*[28]	2017	Amhara	Cross-sectional	378	180 (47.62)	NR	141 (37.30)	91.8%	9

## References

[1] Woldegeorgis BZ, Kerbo AA, Obsa MS, Mokonnon TM. A systematic review and meta-analysis of antimicrobial resistance knowledge, attitudes, and practices: Current evidence to build a strong national antimicrobial drug resistance narrative in Ethiopia. PLoS One. 2023;18(6):e0287042. doi: 10.1371/journal.pone.0287042 37294747 PMC10256206

