# Supplementary material for: A systematic review and meta-analysis of antimicrobial resistance knowledge, attitudes, and practices: Current evidence to build a strong national antimicrobial drug resistance narrative in Ethiopia
Source: PLoS One. 2023 Jun 9;18(6):e0287042. doi: 10.1371/journal.pone.0287042 (PMC10256206; doi:10.1371/journal.pone.0287042)
Supplement: S1 Table — (DOCX) [file pone.0287042.s002.docx]

**S1Table: Search detail and concept map**

| **Concept map 1:**Knowledge | Keywords | Knowledge[Text Word] |
| --- | --- | --- |
|  | MeSH terms | "Knowledge"[Mesh] |
| **Concept map 2:**Attitude | Keywords | "Attitude"[text word] |
|  | MeSH terms | "Attitude"[Mesh] |
| **Concept map 3:**Practice | Keywords | Practice[Text Word] |
|  | MeSH terms | "Health Knowledge, Attitudes, Practice"[Mesh] |
| Concept map 4: Antimicrobial resistance | Keywords | Antimicrobial resistance[Text Word] |
|  | MeSH terms | "Drug Resistance, Microbial"[Mesh] |
| Concept map 5: associated factors | Keywords | associated factors[Text Word] |
|  | MeSH terms | "Risk Factors"[Mesh] |
| **Concept map 6:**Ethiopians | Keywords | Ethiopia (text word) |
|  | MeSH terms | "Ethiopia"[Mesh] |

"knowledge"[MeSH Terms] OR Knowledge[Text Word] AND "attitude"[MeSH Terms] OR Attitude[Text Word] AND Practices[All Fields] AND ("anti-infective agents"[All Fields] OR "anti-infective agents"[MeSH Terms] OR Antimicrobial[Text Word]) AND "Risk Factors"[Mesh] AND "ethiopia"[MeSH Terms] OR Ethiopia[Text Word]
