# Supplementary material for: A systematic review and meta-analysis of antimicrobial resistance knowledge, attitudes, and practices: Current evidence to build a strong national antimicrobial drug resistance narrative in Ethiopia
Source: PLoS One. 2023 Jun 9;18(6):e0287042. doi: 10.1371/journal.pone.0287042 (PMC10256206; doi:10.1371/journal.pone.0287042)
Supplement: S2 Table — (B) Analytical cross-sectional studies. (DOCX) [file pone.0287042.s003.docx]

**S2 Table A** : Quality assessment of studies using JBI’s critical appraisal tools designed for Descriptive cross-sectional study

| Study | Sample size | JBI’s critical appraisal questions | | | | | | | | | Score | Overall Appraisal |
| --- | --- | --- | --- | --- | --- | --- | --- | --- | --- | --- | --- | --- |
|  |  | Q1 | Q2 | Q3 | Q4 | Q5 | Q6 | Q7 | Q8 | Q9 |  |  |
| Bayeh et al. | 385 | Y | Y | Y | Y | y | Y | Y | Y | Y | 9 | Included |
| Belachew et al. | 276 | Y | Y | Y | Y | Y | Y | Y | U | Y | 9 | Included |
| Beyene et al. | 205 | Y | Y | Y | Y | Y | Y | Y | Y | Y | 9 | Included |
| Dejene et al. | 400 | Y | Y | Y | Y | Y | Y | Y | Y | Y | 9 | Included |
| Fetensa et al. | 232 | Y | Y | Y | Y | Y | Y | Y | Y | Y | 9 | Included |
| Gemeda et al. | 379 | Y | Y | Y | Y | Y | Y | Y | Y | Y | 9 | Included |
| Geta and Kibret | 91 | Y | Y | Y | Y | Y | Y | Y | Y | Y | 9 | Included |
| Geta and Kibret | 232 | Y | Y | Y | Y | y | Y | Y | Y | Y | 9 | Included |
| Mengesha et al. | 374 | Y | Y | Y | Y | Y | Y | Y | N | Y | 8 | Included |
| Seid et al. | 323 | Y | Y | Y | Y | Y | Y | Y | Y | Y | 9 | Included |
| Tafa et al | 218 | Y | Y | Y | N | Y | Y | Y | Y | Y | 8 | Included |
| Tesfaye et al | 378 | Y | Y | Y | Y | Y | Y | Y | Y | Y | 9 | Included |

Y –Yes;N-No;U -Unclear-Question. Overall score is calculated by counting the number of Y’s in each row.Q1=Was the sample frame appropriate to address the target population? Q2=Were study participants sampled in an appropriate way? Q3=Was the sample size adequate? Q4=Were the study subjects and the setting described in detail? Q5=Was the data analysis conducted with sufficient coverage of the identified sample? Q6=Were valid methods used for the identification of the condition? Q7=Was the condition measured in a standard, reliable way for all participants? Q8=Was there appropriate statistical analysis? Q9=Was the response rate adequate, and if not, was the low response rate managed appropriately?

**S2 Table B**: Quality assessment of studies using JBI’s critical appraisal tools designed for Analytical cross-sectional study

| Study | Sample size | JBI’s critical appraisal questions | | | | | | | | Score | Overall Appraisal |
| --- | --- | --- | --- | --- | --- | --- | --- | --- | --- | --- | --- |
|  |  | Q1 | Q2 | Q3 | Q4 | Q5 | Q6 | Q7 | Q8 |  |  |
| Gebeyehu et al | 571 | Y | y | y | y | y | y | y | y | 8 | Included |
| Simegn et al | 412 | y | y | y | y | y | y | y | y | 8 | Included |

Y –Yes;N-No;U -Unclear-Question. Overall score is calculated by counting the number of Y’s in

For analytical cross-sectional study, the JBI checklist assessed the following questions

Major components :

1. Were the criteria for inclusion in the sample clearly defined?

2. Were the study subjects and the setting described in detail?

3. Was the exposure measured in a valid and reliable way?

4. Were objective, standard criteria used for measurement of the condition?

5. Were confounding factors identified?

6. Were strategies to deal with confounding factors stated?

7. Were the outcomes measured in a valid and reliable way?

8. Was appropriate statistical analysis used?
